# Supplementary figures and images for: Characterization of hepatitis B viral forms from patient plasma using velocity gradient: Evidence for an excess of capsids in fractions enriched in Dane particles
Source: PLoS One. 2022 Nov 16;17(11):e0272474. doi: 10.1371/journal.pone.0272474 (PMC9668129; doi:10.1371/journal.pone.0272474)

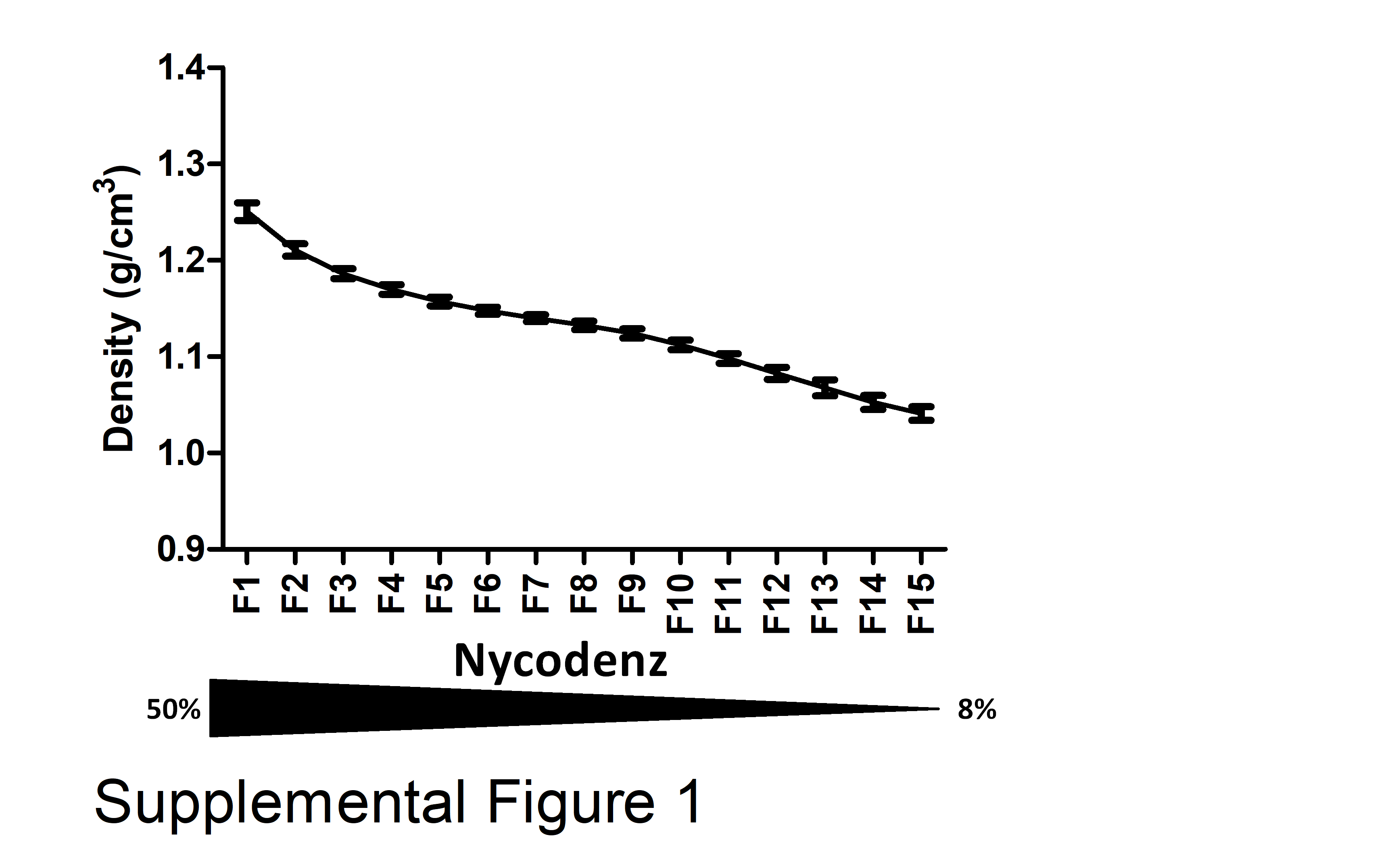

Supplement: S1 Fig — (n = 11, error bars represent standard deviations). (TIF) [file pone.0272474.s001.tif]
