# Supplementary material for: Characterization of hepatitis B viral forms from patient plasma using velocity gradient: Evidence for an excess of capsids in fractions enriched in Dane particles
Source: PLoS One. 2022 Nov 16;17(11):e0272474. doi: 10.1371/journal.pone.0272474 (PMC9668129; doi:10.1371/journal.pone.0272474)
Supplement: S1 File — (DOCX) [file pone.0272474.s002.docx]

**S1 Table**. HBeAg ratios measured with Elecsys® HBeAg assay in each fraction of Nycodenz velocity gradients for 2 plasmas. Positive results are indicated in bold.

|  | HBeAg ratio with Cobas assay  (≥1 = reactive) | |
| --- | --- | --- |
| Fraction | B7505 plasma  (Gt E HBeAg+) | B7686 plasma  (Gt D HBeAg -) |
| 1 | 0.215 | 0.057 |
| 2 | 0.219 | 0.061 |
| 3 | 0.231 | 0.067 |
| 4 | 0.234 | 0.07 |
| 5 | 0.255 | 0.075 |
| 6 | 0.264 | 0.081 |
| 7 | 0.273 | 0.087 |
| 8 | 0.285 | 0.086 |
| 9 | 0.294 | 0.084 |
| 10 | 0.342 | 0.093 |
| 11 | 0.393 | 0.097 |
| 12 | **1.467** | 0.095 |
| 13 | **33.63** | 0.102 |
| 14 | **321** | 0.096 |
| 15 | **1080.3** |  |

**S2 Table**. Raw data of each marker in each fraction of Nycodenz velocity gradient for **B7195 plasma** (Genotype D; HBeAg+)

| Fraction | HBV-DNA (IU/mL) | HBV-RNA (U/mL) | HBsAg (IU/mL) | HBcrAg (logU/mL) |
| --- | --- | --- | --- | --- |
| 1 | 3.55E+04 | 0.00E+00 | 0 | 3 |
| 2 | 9.30E+05 | 2.38E+02 | 0 | 4.2 |
| 3 | 1.67E+07 | 8.21E+03 | 0.9 | 5.6 |
| 4 | 1.18E+08 | 3.20E+04 | 4.8 | 6.5 |
| 5 | 8.94E+07 | 2.54E+04 | 4.9 | 6.5 |
| 6 | 2.52E+07 | 5.86E+03 | 18 | 6.1 |
| 7 | 9.79E+06 | 3.06E+03 | 23 | 5.7 |
| 8 | 4.10E+06 | 9.44E+02 | 51 | 5.2 |
| 9 | 1.92E+06 | 6.40E+02 | 200 | 5.1 |
| 10 | 1.98E+06 | 2.43E+02 | 490 | 5.2 |
| 11 | 1.45E+06 | 3.09E+02 | 980 | 5.4 |
| 12 | 7.36E+05 | 1.94E+02 | 900 | 6.3 |
| 13 | 5.25E+05 | 1.32E+02 | 280 | 6.6 |
| 14 | 3.81E+05 | 0.00E+00 | 89 | 6.9 |
| 15 | 4.18E+05 | 0.00E+00 | 120 | 6.7 |

**S3 Table**. Raw data of each marker in each fraction of Nycodenz velocity gradient for **B7505 plasma** (Genotype E; HBeAg+)

| Fraction | HBV-DNA (IU/mL) | HBV-RNA (U/mL) | HBsAg (IU/mL) | HBcrAg (logU/mL) |
| --- | --- | --- | --- | --- |
| 1 | 8.27E+04 | 3.19E+01 | 0 | 6.2 |
| 2 | 7.30E+04 | 1.65E+03 | 0 | 6.2 |
| 3 | 3.34E+06 | 2.89E+03 | 2.5 | 6.5 |
| 4 | 2.35E+06 | 2.25E+03 | 2.1 | 6.4 |
| 5 | 6.87E+07 | 4.97E+03 | 2.8 | 6.6 |
| 6 | 1.61E+07 | 1.65E+03 | 3.8 | 6.7 |
| 7 | 3.47E+06 | 3.99E+02 | 6.4 | 6.3 |
| 8 | 6.34E+05 | 1.88E+02 | 17 | 5.7 |
| 9 | 5.02E+05 | 5.32E+01 | 48 | 5.3 |
| 10 | 7.94E+03 | 5.15E+01 | 100 | 5.2 |
| 11 | 3.62E+04 | 4.86E+01 | 300 | 5.3 |
| 12 | 2.63E+04 | 3.13E+01 | 1200 | 5.8 |
| 13 | 1.64E+05 | 4.20E+01 | 1663 | 6.2 |
| 14 | 5.76E+04 | 5.09E+01 | 1598 | 6.6 |
| 15 | 6.41E+04 | 4.93E+01 | 670 | 7.2 |

**S4 Table**. Raw data of each marker in each fraction of Nycodenz velocity gradient for **B7686 plasma** (Genotype D; HBeAg-)

| Fraction | HBV-DNA (IU/mL) | HBV-RNA (U/mL) | HBsAg (IU/mL) | HBcrAg (logU/mL) |
| --- | --- | --- | --- | --- |
| 1 | 0.00E+00 | 0.00E+00 | 0 | 2.8 |
| 2 | 0.00E+00 | 0.00E+00 | 0 | 3 |
| 3 | 2.78E+03 | 0.00E+00 | 0 | 3.1 |
| 4 | 4.05E+04 | 0.00E+00 | 0.8 | 3.7 |
| 5 | 4.71E+04 | 0.00E+00 | 2.3 | 3.8 |
| 6 | 3.30E+04 | 0.00E+00 | 6.6 | 3.6 |
| 7 | 5.10E+03 | 0.00E+00 | 16 | 3.3 |
| 8 | 2.98E+03 | 0.00E+00 | 42 | 3.1 |
| 9 | 1.04E+03 | 0.00E+00 | 93 | 3.1 |
| 10 | 1.90E+03 | 0.00E+00 | 150 | 3.1 |
| 11 | 3.57E+01 | 0.00E+00 | 370 | 3.5 |
| 12 | 0.00E+00 | 0.00E+00 | 570 | 4 |
| 13 | 4.82E+02 | 0.00E+00 | 260 | 4.3 |
| 14 | 0.00E+00 | 0.00E+00 | 82 | 4.3 |

**S5 Table**. Raw data of each marker in each fraction of Nycodenz velocity gradient for **B7207 plasma** (Genotype E; HBeAg-)

| Fraction | HBV-DNA (IU/mL) | HBV-RNA (U/mL) | HBsAg (IU/mL) | HBcrAg (logU/mL) |
| --- | --- | --- | --- | --- |
| 1 | 1.76E+04 | 1.61E+01 | 0.3 | 3.9 |
| 2 | 8.48E+05 | 3.11E+02 | 0.3 | 5.1 |
| 3 | 3.26E+06 | 1.31E+03 | 1.6 | 5.7 |
| 4 | 5.77E+06 | 2.43E+03 | 5.4 | 6.1 |
| 5 | 6.86E+06 | 1.93E+03 | 17 | 6.1 |
| 6 | 1.05E+06 | 9.16E+02 | 45 | 5.9 |
| 7 | 7.36E+05 | 2.62E+02 | 83 | 5.5 |
| 8 | 1.18E+05 | 1.30E+02 | 110 | 5.1 |
| 9 | 1.07E+05 | 1.27E+02 | 140 | 5 |
| 10 | 1.63E+05 | 1.25E+02 | 220 | 4.9 |
| 11 | 4.76E+05 | 1.34E+02 | 670 | 5.2 |
| 12 | 6.80E+04 | 1.39E+02 | 980 | 4.9 |
| 13 | 7.05E+04 | 3.34E+01 | 530 | 4.6 |
| 14 | 2.64E+04 | 6.56E+01 | 200 | 4.5 |

**S6 Table**. Raw data of each marker in each fraction of Nycodenz velocity gradient for **B7195 plasma** (Genotype D; HBeAg+) treated with **NP-40** before fractionation.

| Fraction | HBV-DNA (IU/mL) | HBV-RNA (U/mL) | HBsAg (IU/mL) | HBcrAg (logU/mL) |
| --- | --- | --- | --- | --- |
| 1 | 1.05E+08 | 3.13E+02 | 0 | 6.9 |
| 2 | 4.50E+07 | 2.12E+02 | 0 | 6.4 |
| 3 | 1.33E+07 | 6.40E+01 | 0 | 5.8 |
| 4 | 4.57E+06 | 2.81E+01 | 0 | 5.3 |
| 5 | 2.31E+06 | 1.74E+01 | 0.5 | 4.9 |
| 6 | 1.41E+06 | 0.00E+00 | 1.4 | 4.8 |
| 7 | 1.06E+06 | 0.00E+00 | 6.3 | 4.6 |
| 8 | 5.03E+05 | 0.00E+00 | 32 | 4.5 |
| 9 | 7.12E+05 | 0.00E+00 | 110 | 4.6 |
| 10 | 6.80E+05 | 0.00E+00 | 610 | 5.1 |
| 11 | 8.73E+05 | 0.00E+00 | 960 | 5.5 |
| 12 | 7.50E+05 | 0.00E+00 | 1300 | 6.3 |
| 13 | 4.51E+05 | 0.00E+00 | 960 | 6.7 |
| 14 | 3.88E+05 | 0.00E+00 | 720 | 7 |
| 15 | 7.52E+05 | 0.00E+00 | 740 | 6.8 |

**S7 Table**. Raw data of each marker in each fraction of Nycodenz velocity gradient for **B7207 plasma** (Genotype E; HBeAg-) treated with **NP-40** before fractionation.

| Fraction | HBV-DNA (IU/mL) | HBV-RNA (U/mL) | HBsAg (IU/mL) | HBcrAg (logU/mL) |
| --- | --- | --- | --- | --- |
| 1 | 7.66E+07 | 8.81E+02 | 0 | 6.1 |
| 2 | 1.51E+07 | 2.29E+02 | 0 | 5.7 |
| 3 | 7.24E+06 | 7.85E+01 | 0 | 5.3 |
| 4 | 2.56E+06 | 3.17E+01 | 0.9 | 4.9 |
| 5 | 1.85E+06 | 3.05E+01 | 2 | 4.6 |
| 6 | 1.88E+06 | 2.55E+01 | 3.1 | 4.7 |
| 7 | 1.18E+06 | 2.81E+01 | 4.6 | 4.5 |
| 8 | 8.89E+05 | 0.00E+00 | 8.6 | 4.5 |
| 9 | 1.81E+06 | 0.00E+00 | 18 | 4.6 |
| 10 | 2.88E+06 | 1.77E+01 | 35 | 4.8 |
| 11 | 4.62E+06 | 2.67E+01 | 260 | 5.2 |
| 12 | 3.23E+06 | 2.12E+01 | 880 | 5.1 |
| 13 | 1.98E+06 | 2.23E+01 | 1440 | 4.7 |
| 14 | 9.23E+05 | 0.00E+00 | 740 | 4.5 |
| 15 | 5.65E+05 | 0.00E+00 | 690 | 4.5 |
| 16 | 1.06E+06 | 1.25E+01 | 690 | 4.5 |

**S8 Table**. Raw data of each marker in each fraction of sucrose density gradient for **B7195 plasma** (Genotype D; HBeAg+).

| Fraction | HBV-DNA (IU/mL) | HBV-RNA (U/mL) | HBsAg (IU/mL) | HBcrAg (logU/mL) |
| --- | --- | --- | --- | --- |
| 1 | 1.52E+05 | 1.30E+01 | 0 | 5.3 |
| 2 | 1.10E+06 | 1.14E+02 | 0 | 4.5 |
| 3 | 9.06E+06 | 5.75E+02 | 0 | 5.4 |
| 4 | 6.42E+07 | 5.94E+03 | 1.6 | 6.2 |
| 5 | 1.92E+08 | 2.45E+04 | 8 | 6.6 |
| 6 | 9.63E+07 | 1.84E+04 | 14 | 6.6 |
| 7 | 3.67E+07 | 7.67E+03 | 45 | 6.4 |
| 8 | 2.21E+07 | 4.60E+03 | 120 | 6.1 |
| 9 | 9.44E+06 | 2.17E+03 | 250 | 5.9 |
| 10 | 8.93E+06 | 1.21E+03 | 640 | 5.8 |
| 11 | 6.08E+06 | 3.22E+02 | 1100 | 6 |
| 12 | 3.60E+06 | 2.52E+02 | 1000 | 6.2 |
| 13 | 2.80E+06 | 2.19E+02 | 940 | 6.7 |
| 14 | 1.98E+06 | 1.73E+02 | 620 | 6.7 |
| 15 | 1.44E+06 | 7.50E+01 | 280 | 6.8 |
| 16 | 2.87E+06 | 5.75E+02 | 230 | 6.7 |

**S9 Table**. Raw data of each marker in each fraction of sucrose density gradient for **B7207 plasma** (Genotype E; HBeAg-).

| Fraction | HBV-DNA (IU/mL) | HBV-RNA (U/mL) | HBsAg (IU/mL) | HBcrAg (logU/mL) |
| --- | --- | --- | --- | --- |
| 1 | 1.24E+05 | 4.34E+01 | 0 | 3.7 |
| 2 | 4.66E+05 | 5.39E+01 | 0 | 4.1 |
| 3 | 1.17E+06 | 1.70E+02 | 0 | 4.5 |
| 4 | 3.08E+06 | 3.72E+02 | 0 | 5 |
| 5 | 1.09E+07 | 1.52E+03 | 0.8 | 5.6 |
| 6 | 2.34E+07 | 4.48E+03 | 1.9 | 6.2 |
| 7 | 1.52E+07 | 6.02E+03 | 14 | 6.3 |
| 8 | 1.90E+06 | 2.31E+03 | 96 | 6.1 |
| 9 | 5.00E+06 | 1.15E+03 | 170 | 5.7 |
| 10 | 2.94E+06 | 8.64E+02 | 430 | 5.5 |
| 11 | 1.23E+06 | 4.18E+02 | 850 | 5.3 |
| 12 | 5.74E+05 | 1.51E+02 | 810 | 4.8 |
| 13 | 3.19E+05 | 6.12E+01 | 470 | 5.1 |
| 14 | 3.76E+05 | 3.42E+01 | 260 | 4.6 |
| 15 | 6.91E+05 | 1.78E+02 | 110 | 4.8 |

**S10 Table**. Raw data of each marker in each fraction of sucrose density gradient for **B7505 plasma** (Genotype E; HBeAg+) treated with **NP-40** before fractionation.

| Fraction | HBV-DNA (IU/mL) | HBV-RNA (U/mL) | HBsAg (IU/mL) | HBcrAg (logU/mL) |
| --- | --- | --- | --- | --- |
| 1 | 4.00E+07 | 4.57E+03 | 0 | 5.2 |
| 2 | 7.57E+07 | 3.82E+03 | 0 | 5.7 |
| 3 | 1.72E+07 | 8.21E+02 | 0 | 5.7 |
| 4 | 8.88E+06 | 1.40E+03 | 0 | 5.8 |
| 5 | 1.03E+07 | 5.78E+02 | 0.0 | 5.5 |
| 6 | 2.29E+06 | 5.28E+02 | 0.0 | 5 |
| 7 | 3.92E+06 | 0.00E+00 | 0 | 5 |
| 8 | 1.24E+06 | 0.00E+00 | 0 | 4.7 |
| 9 | 1.25E+06 | 0.00E+00 | 1 | 4.5 |
| 10 | 6.72E+05 | 0.00E+00 | 1.6 | 4.5 |
| 11 | 1.27E+06 | 0.00E+00 | 410 | 4.7 |
| 12 | 1.67E+06 | 0.00E+00 | 1847 | 5.5 |
| 13 | 1.42E+06 | 0.00E+00 | 2035 | 5.6 |
| 14 | 1.05E+06 | 0.00E+00 | 2022 | 5.8 |
| 15 | 1.02E+06 | 0.00E+00 | 1933 | 6 |
| 16 | 1.17E+06 | 0.00E+00 | 1514 | 6 |

**S11 Table**. Raw data of each marker in each fraction of sucrose density gradient for **B7207 plasma** (Genotype E; HBeAg-) treated with **NP-40** before fractionation.

| Fraction | HBV-DNA (IU/mL) | HBV-RNA (U/mL) | HBsAg (IU/mL) | HBcrAg (logU/mL) |
| --- | --- | --- | --- | --- |
| 1 | 5.68E+07 | 1.93E+04 | 0 | 5.2 |
| 2 | 3.36E+07 | 2.87E+04 | 0 | 5.2 |
| 3 | 1.29E+07 | 1.13E+04 | 0.4 | 4.9 |
| 4 | 9.09E+06 | 3.17E+03 | 0.6 | 4.6 |
| 5 | 3.20E+06 | 1.91E+03 | 2.0 | 4.3 |
| 6 | 2.84E+06 | 1.29E+03 | 11.0 | 4.2 |
| 7 | 1.60E+06 | 9.50E+02 | 37 | 4.1 |
| 8 | 1.07E+06 | 0.00E+00 | 79 | 3.9 |
| 9 | 8.69E+05 | 0.00E+00 | 180 | 3.9 |
| 10 | 5.23E+05 | 0.00E+00 | 480 | 3.8 |
| 11 | 2.23E+05 | 6.73E+02 | 850 | 3.9 |
| 12 | 1.85E+05 | 0.00E+00 | 1000 | 3.8 |
| 13 | 8.76E+04 | 0.00E+00 | 1100 | 3.8 |
| 14 | 8.46E+04 | 0.00E+00 | 990 | 3.7 |
| 15 | 4.89E+05 | 5.39E+02 | 740 | 3.7 |

**S12 Table**. Raw data of each marker in each fraction of sucrose density gradient of the most DNA enriched fractions from Nycodenz velocity gradient for **B7505 plasma** (Genotype E; HBeAg+).

| Fraction | HBV-DNA (IU/mL) | HBV-RNA (U/mL) | HBsAg (IU/mL) | HBcrAg (logU/mL) |
| --- | --- | --- | --- | --- |
| 1 | 2.09E+04 | 0.00E+00 | 3 | 2.8 |
| 2 | 5.28E+04 | 0.00E+00 | 11 | 3.2 |
| 3 | 4.45E+05 | 0.00E+00 | 49 | 3.7 |
| 4 | 2.09E+06 | 0.00E+00 | 191 | 4.4 |
| 5 | 3.28E+06 | 0.00E+00 | 435 | 4.7 |
| 6 | 2.09E+06 | 0.00E+00 | 342 | 4.6 |
| 7 | 1.25E+06 | 0.00E+00 | 174 | 4.3 |
| 8 | 4.82E+05 | 0.00E+00 | 133 | 4 |
| 9 | 3.92E+05 | 0.00E+00 | 100 | 3.8 |
| 10 | 2.42E+05 | 0.00E+00 | 64 | 3.7 |
| 11 | 2.10E+05 | 0.00E+00 | 34 | 3.4 |
| 12 | 1.28E+05 | 0.00E+00 | 18 | 3.2 |
| 13 | 1.62E+05 | 0.00E+00 | 14 | 3.2 |
| 14 | 1.35E+05 | 0.00E+00 | 25 | 3.4 |

**S13 Table**. Raw data of each marker in each fraction of sucrose density gradient of the most DNA enriched fraction from Nycodenz velocity gradient for **B7207** **plasma** (Genotype E; HBeAg-).

| Fraction | HBV-DNA (IU/mL) | HBV-RNA (U/mL) | HBsAg (IU/mL) | HBcrAg (logU/mL) |
| --- | --- | --- | --- | --- |
| 1 | 0.00E+00 | 0.00E+00 | 1 | 2 |
| 2 | 0.00E+00 | 0.00E+00 | 1 | 2 |
| 3 | 4.95E+03 | 0.00E+00 | 3 | 2.5 |
| 4 | 3.16E+03 | 0.00E+00 | 4 | 2.6 |
| 5 | 4.11E+04 | 9.50E+02 | 20 | 3.7 |
| 6 | 7.93E+05 | 3.28E+03 | 136 | 4.3 |
| 7 | 1.71E+06 | 2.16E+03 | 575 | 4.4 |
| 8 | 6.59E+05 | 1.62E+03 | 1816 | 4.2 |
| 9 | 4.68E+05 | 8.92E+02 | 2222 | 3.9 |
| 10 | 2.36E+05 | 6.73E+02 | 1288 | 3.6 |
| 11 | 1.38E+05 | 0.00E+00 | 630 | 3.2 |
| 12 | 7.61E+04 | 0.00E+00 | 330 | 3.1 |
| 13 | 7.93E+04 | 0.00E+00 | 153 | 3 |
| 14 | 5.07E+04 | 0.00E+00 | 87 | 3 |
| 15 | 4.09E+04 | 0.00E+00 | 49 | 2.9 |
| 16 | 7.47E+04 | 0.00E+00 | 83 | 3 |

**S14 Table**. Number of Virions particles (VP) for **B7505** plasma (Genotype E; HBeAg+), calculated according to DNA (VP_DNA_), HBc (VP_HBc_) or HBs (VP_HBs_) concentration per fraction after a 30–60% sucrose density gradient from the richest DNA fractions obtained from velocity gradients.

| Fraction | VPDNA / Fraction | VPHBs / Fraction | VPHBc / Fraction |
| --- | --- | --- | --- |
| 1 | 2.61E+04 | 1.36E+05 | 0.00E+00 |
| 2 | 6.60E+04 | 4.99E+05 | 4.49E+06 |
| 3 | 5.56E+05 | 2.22E+06 | 1.42E+07 |
| 4 | 2.61E+06 | 8.66E+06 | 7.12E+07 |
| 5 | 4.10E+06 | 1.97E+07 | 1.42E+08 |
| 6 | 2.61E+06 | 1.55E+07 | 1.13E+08 |
| 7 | 1.56E+06 | 7.89E+06 | 5.65E+07 |
| 8 | 6.03E+05 | 6.03E+06 | 2.83E+07 |
| 9 | 4.90E+05 | 4.54E+06 | 1.79E+07 |
| 10 | 3.03E+05 | 2.90E+06 | 1.42E+07 |
| 11 | 2.63E+05 | 1.54E+06 | 7.12E+06 |
| 12 | 1.60E+05 | 8.16E+05 | 4.49E+06 |
| 13 | 2.03E+05 | 6.35E+05 | 4.49E+06 |
| 14 | 1.69E+05 | 1.13E+06 | 7.12E+06 |

**S15 Table**. Number of Virions particles (VP) for **B7207 plasma** (Genotype E; HBeAg-), calculated according to DNA (VP_DNA_), HBc (VP_HBc_) or HBs (VP_HBs_) concentration per fraction after a 30–60% sucrose density gradient from the richest DNA fractions obtained from velocity gradients.

| Fraction | VPDNA / Fraction | VPHBs / Fraction | VPHBc / Fraction |
| --- | --- | --- | --- |
| 1 | 0.00E+00 | 4.54E+04 | 2.83E+05 |
| 2 | 0.00E+00 | 4.54E+04 | 2.83E+05 |
| 3 | 6.19E+03 | 1.36E+05 | 8.96E+05 |
| 4 | 3.95E+03 | 1.81E+05 | 1.13E+06 |
| 5 | 5.14E+04 | 9.07E+05 | 1.42E+07 |
| 6 | 9.91E+05 | 6.17E+06 | 5.65E+07 |
| 7 | 2.14E+06 | 2.61E+07 | 7.12E+07 |
| 8 | 8.24E+05 | 8.24E+07 | 4.49E+07 |
| 9 | 5.85E+05 | 1.01E+08 | 2.25E+07 |
| 10 | 2.95E+05 | 5.84E+07 | 1.13E+07 |
| 11 | 1.73E+05 | 2.86E+07 | 4.49E+06 |
| 12 | 9.51E+04 | 1.50E+07 | 3.57E+06 |
| 13 | 9.91E+04 | 6.94E+06 | 2.83E+06 |
| 14 | 6.34E+04 | 3.95E+06 | 2.83E+06 |
| 15 | 5.11E+04 | 2.22E+06 | 2.25E+06 |
| 16 | 9.34E+04 | 3.76E+06 | 2.83E+06 |
