# Supplementary material for: Characterization of hepatitis B viral forms from patient plasma using velocity gradient: Evidence for an excess of capsids in fractions enriched in Dane particles
Source: PLoS One. 2022 Nov 16;17(11):e0272474. doi: 10.1371/journal.pone.0272474 (PMC9668129; doi:10.1371/journal.pone.0272474)
Supplement: Attachment — Submitted filename: gradient centrifugation of plasm samples.docx [file pone.0272474.s003.docx]

The authors analyzed 4 plasma samples (genotype D or E, HBeAg+ or HBeAg-) by nycodenz or sucrose gradient centrifugation, or sequential centrifugation for better separation, with or with NP40 treatment to convert virions into capsids. The purified fractions were analyzed for HBV DNA, HBV RNA, HBcrAg, and HBsAg. Based on their measurements and calculation, they suggested presence of enveloped core particles (virions) lacking HBV DNA or RNA (empty virions; previously reported by Jianming Hu’s lab), enveloped core particles containing HBV RNA (reported before by many groups), in addition to enveloped core particles lacking envelope protein (“budding capsids”; never described before). They also found HBcrAg in fractions lighter than subviral particles even for HBeAg-negative samples, which they attributed to immune complex between HBeAg and anti-HBe. They also calculated that for HBeAg-positive samples, HBeAg accounts for 2/3rds of HBcrAg. In general, this was an interesting study. Some questions:

1. Were all the four plasma samples well preserved? No issue of poor storage and sample degradation?
2. I am not sure if the calculations and conversion of the amount of DNA or protein into capsids, virions, subviral particles are correct (Does one virion contain 180 or 240 copies of core protein? Does it contain 1 or 2 genomes?).
3. The huge excess of subviral particles relative to virions, which become even more serious for the HBeAg-negative samples, may cause contamination by subviral particles in virion-enriched fractions. The latter point was confirmed in Fig. 5, by further centrifugation of a virion-enriched fraction from nycodenz gradient through sucrose cushion. That point is also suggested by the much higher VPHBs/VPDNA ratio and much lower VPHBc/VPHBs ratio from the two HBeAg-negative samples (especially the genotype D sample; B7686) than the two HBeAg-positive samples. Thus, I would tend to more believe in data generated from the HBeAg-positive samples.
4. As the authors discussed, the presence of “budding capsids” requires validation from electron microscopy. Alternatively, zycodenz gradient followed by sucrose gradient centrifugation can be further followed by native agarose gel electrophoresis (NAGE), with subsequent detection of HBcrAg, HBsAg, and HBV DNA. That point can be raised in the Discussion section.

Minor issues: English writing and clarity of presentation need improvement. For example, “in Dane particles enriched fractions” in the title should be “in fractions enriched in Dane particles”. Lines 49-50: “hepadnaviridae family……..gathers DNA reverse transcribing hepatotropic viruses”. “gather” should be changed.
